# Bidirectional relation between depression and sudden sensorineural hearing loss: Two longitudinal follow-up studies using a national sample cohort

**DOI:** 10.1038/s41598-020-58547-w

**Published:** 2020-01-30

**Authors:** So Young Kim, Chanyang Min, Chang Ho Lee, Bumjung Park, Hyo Geun Choi

**Affiliations:** 1 Department of Otorhinolaryngology-Head & Neck Surgery, CHA Bundang Medical Center, CHA University, Seongnam, Korea; 2 Hallym Data Science Laboratory, Hallym University College of Medicine, Anyang, Korea; 3 Graduate School of Public Health, Seoul National University, Seoul, Korea; 4 Department of Otorhinolaryngology-Head & Neck Surgery, Hallym University College of Medicine, Anyang, Korea

**Keywords:** Epidemiology, Risk factors

## Abstract

A few studies have explored the association between depression and sudden sensorineural hearing loss (SSNHL). This study was aimed to investigate the reciprocal relations between SSNHL and depression using a nationwide cohort of the Korean population. Subjects aged > 20 years from the Korean National Health Insurance Service-National Sample Cohort were enrolled from 2002 to 2013. In study I, a total of 60,178 depressed patients were matched 1:4 with 242,872 control I subjects. In study II, a total of 4,328 SSNHL patients were 1:4 matched with 17,312 control II subjects. They were matched for age, sex, income, and region of residence. The Charlson comorbidity index (CCI) was adjusted. Subgroup analysis was performed according to age and sex. The crude and adjusted CCI hazard ratios (HRs) of SSNHL in depressed patients (study I) and depression in SSNHL patients (study II) were analyzed using the stratified Cox proportional-hazard model. In study I, the depression group exhibited an elevated adjusted HR of SSNHL 1.16 times that of the control group (confidence interval [95% CI] = 1.02–1.31, P < 0.023). The middle-aged women subgroup demonstrated an increased risk of SSNHL within the depression group. In study II, the SSNHL group showed a higher adjusted HR of depression 1.29 times that of control II group (95% CI = 1.06–1.57, P = 0.010). The middle-aged women subgroup showed an elevated risk of depression in the SSNHL group. The risk of SSNHL was elevated in the depressed patients and the risk of depression was increased in the SSNHL patients.

## Introduction

Depression is a pathologic condition involving feelings of sadness and a loss of interest in activities that last at least two weeks. Several psychiatric diseases including major depressive disorders, dysthymic disorder and depressive disorder not otherwise specified are categorized as depressive disorder^1^. The lifetime prevalence of depression is as high as 20–25% in women and 7–12% in men in the general population^2^. In Korea, 9.6–17.3% and 2.8–3.6% of adults complain of depressive symptoms and depression, respectively^3^. The multiple factors of genetic or external factors were suggested to cause depression by evoking inflammation of the nervous system and subsequent vulnerability to social stress^4^. Cardiovascular and metabolic disorders could be risk factors for depression^5–7^. In addition, social isolation and loneliness were reported to elevate the risk of depressive symptoms^8^. Additionally, it was suggested that impaired social networks due to hearing loss might increase the risk of depression^9^.

Sensorineural hearing loss of ≥30 dB at 3 consecutive frequencies within 3 days of onset is defined as sudden sensorineural hearing loss (SSNHL). Approximately 27 per 100,000 people in the United States experience SSNHL annually^10^. The prevalence of SSNHL is 15 per 100,000 people in Korea^11^. The pathophysiologic causes of SSNHL have not yet been fully explained. Several etiologies associated with immunologic, ischemic, and endocrine factors have been proposed^12–14^. In addition to associations with chronic diseases such as hypertension, hypercholesteremia, and diabetes, recent studies have demonstrated a relation between SSNHL and depression^15,16^.

The impaired social communication could increase the risk of social isolation and depression. Especially, SSNHL patients could experience much frustration, because their hearing ability was normal before onset of SSNHL. Indeed several previous studies reported the high rate of depression in SSNHL patients^16^. A population cohort study reported the risk of depression following SSNHL^16^. On the other hands, the increased inflammation of nervous system and related comorbidities in depression patients could increase the risk of SSNHL. In line with this, one recent study reported the risk of SSNHL in depressed patients using a population-based cohort^15^. Thus, it could be inferred that the relation between depression and SSNHL might be bidirectional. The working hypothesis of the present study was that depression and SSNHL have reciprocal relations. No prior study investigated the bidirectional association between SSNHL and depression. We analyzed this relation using the merits of cohort data by time sequence.

## Results

### Study I

The time duration from index date to SSNHL was 49.1 months (SD = 35.2) in the depression group and 52.8 months (SD = 36.2) in the control group. Age, sex, income, and region of residence were matched between the depression and control groups (Table 1). The incidence of SSNHL was higher in the depression group (0.6% [371/60,718]) than in the control group (0.5% [1,160/242,872], P < 0.001). The CCI was different between the depression and control I groups (P < 0.001).

**Table 1:** General Characteristics of Participants.

Characteristics	Study I			Study II		
	Depression (n, %)	Control I (n, %)	P-value	SSNHL (n, %)	Control II (n, %)	P-value
Age (years old)			1.000			1.000
20–24	3,850 (6.3)	15,400 (6.3)		141 (3.3)	564 (3.3)	
25–29	4,546 (7.5)	18,184 (7.5)		238 (5.5)	952 (5.5)	
30–34	5,236 (8.6)	20,944 (8.6)		271 (6.3)	1,084 (6.3)	
35–39	5,791 (9.5)	23,164 (9.5)		357 (8.3)	1,428 (8.3)	
40–44	6,161 (10.1)	24,644 (10.1)		419 (9.7)	1,676 (9.7)	
45–49	6,446 (10.6)	25,784 (10.6)		457 (10.6)	1,828 (10.6)	
50–54	6,186 (10.2)	24,744 (10.2)		553 (12.8)	2,212 (12.8)	
55–59	5,033 (8.3)	20,132 (8.3)		525 (12.1)	2,100 (12.1)	
60–64	4,712 (7.8)	18,848 (7.8)		444 (10.3)	1,776 (10.3)	
65–69	4,570 (7.5)	18,280 (7.5)		389 (9.0)	1,556 (9.0)	
70–74	3,840 (6.3)	15,360 (6.3)		291 (6.7)	1,164 (6.7)	
75–79	2,431 (4.0)	9,724 (4.0)		160 (3.7)	640 (3.7)	
80–84	1,274 (2.1)	5,096 (2.1)		57 (1.3)	228 (1.3)	
85+	642 (1.1)	2,568 (1.1)		26 (0.6)	104 (0.6)	
Sex		1.000			1.000	
Male	20,705 (34.1)	82,820 (34.1)		1,976 (45.7)	7,904 (45.7)	
Female	40,013 (65.9)	160,052 (65.9)		2,352 (54.3)	9,408 (54.3)	
Income			1.000			1.000
1 (lowest)	9,521 (15.7)	38,084 (15.7)		637 (14.7)	2,548 (14.7)	
2	8,962 (14.8)	35,848 (14.8)		559 (12.9)	2,236 (12.9)	
3	10,448 (17.2)	41,792 (17.2)		755 (17.4)	3,020 (17.4)	
4	13,063 (21.5)	52,252 (21.5)		967 (22.3)	3,868 (22.3)	
5 (highest)	18,724 (30.8)	74,896 (30.8)		1,410 (32.6)	5,640 (32.6)	
Region of residence			1.000			1.000
Urban	27,771 (45.7)	111,084 (45.7)		2,036 (47.0)	8,144 (47.0)	
Rural	32,947 (54.3)	131,788 (54.3)		2,292 (53.0)	9,168 (53.0)	
CCI (score)			<0.001*			<0.001*
0	17,976 (29.6)	104,740 (43.1)		1,196 (27.6)	7,416 (42.8)	
1	5,809 (9.6)	25,616 (10.5)		398 (9.2)	1,616 (9.3)	
≥2	36,933 (60.8)	112,516 (46.3)		2,734 (63.2)	8,280 (47.8)	
SSNHL	371 (0.6)	1,160 (0.5)	<0.001*	4,328 (100.0)	0 (0.0)	<0.001*
Depression	60,718 (100.0)	0 (0.0)	<0.001*	332 (7.7)	993 (5.7)	<0.001*

*Chi-square test, Significance at P Ch0.05

Abbreviation: CCI, Charlson Comorbidity Index; SSNHL, Sudden sensory neural hearing loss.

**Figure 1:**
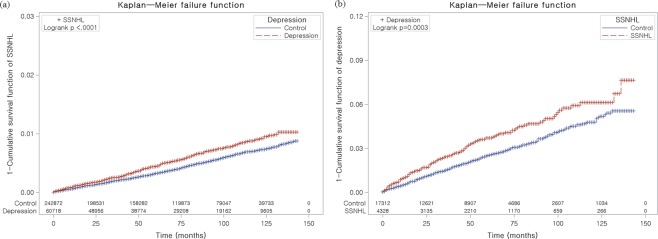
Kaplan-Meier survival analysis. (**a**) The cumulative rate of SSNHL was higher in the depression group than in the control I group; (**b**) The group with SSNHL demonstrated a higher cumulative rate of depression than the control group.

Depression increased the HR of SSNHL (adjusted HR = 1.16, 95% CI = 1.02–1.31; P = 0.023) (Table 2 and Fig. 1a). The depression with ≥ 4 times of treatments were also increased in SSNHL group compared to control I group (adjusted HR = 1.19, 95% CI = 1.04–1.36, P = 0.011, S1 Table).

**Table 2:** Crude and adjusted hazard ratios (95% confidence interval) of SSNHL in depression (study I) and depression in SSNHL (study II).

Characteristics	HRs			
	Crude^†^	P-value	Adjusted†‡	P-value
Study I				
Depression	1.29 (1.14–1.47)	<0.001*	1.16 (1.02–1.31)	0.023*
Control	1.00		1.00	
Study II				
SSNHL	1.42 (1.17–1.72)	<0.001*	1.29 (1.06–1.57)	0.010*
Control	1.00		1.00	

*Cox-proportional hazard regression model, Significance at P Co0.05.

^†^Stratified model for age, sex, income, and region of residence.

^‡^Adjusted model for Charlson Comorbidity Index.

In the subgroup analysis according to age and sex, middle-aged women showed high HRs for SSNHL (adjusted HR = 1.36, 95% CI = 1.10–1.68, P = 0.005) (Table 3). The crude and adjusted HRs did not reach statistical significance in either the young or old men/women.

**Table 3:** Subgroup analysis of crude and adjusted hazard ratios (95% confidence interval) of sudden sensory neural hearing loss in depression according to age and sex in study I.

Characteristics	HRs of SSNHL			
	Crude^†^	P-value	Adjusted^†‡^	P-value
**Young men (20–39 years old, n = 33,400)**				
Depression	1.17 (0.71–1.93)	0.531	0.99 (0.60–1.64)	0.967
Control I	1.00		1.00	
**Young women (20–39 years old, n = 63,715)**				
Depression	1.20 (0.85–1.70)	0.311	1.08 (0.76–1.53)	0.688
Control I	1.00		1.00	
**Middle aged men (40–59 years old, n = 40,510)**				
Depression	1.39 (1.00–1.92)	0.047*	1.22 (0.88–1.70)	0.229
Control I	1.00		1.00	
**Middle aged women (40–59 years old, n = 78,620)**				
Depression	1.54 (1.25–1.90)	<0.001*	1.36 (1.10–1.68)	0.005*
Control I	1.00		1.00	
**Old men (60 + years old, n = 29,615)**				
Depression	1.05 (0.69–1.58)	0.832	0.92 (0.61–1.40)	0.709
Control I	1.00		1.00	
**Old women (60 + years old, n = 57,730)**				
Depression	1.24 (0.86–1.47)	0.386	1.03 (0.79–1.34)	0.836
Control I	1.00		1.00	

*Cox-proportional hazard regression model, Significance at P Co0.05.

^†^Stratified model for age, sex, income, and region of residence.

^‡^Adjusted model for Charlson Comorbidity Index.

### Study II

The time duration from index date to depression was 34.9 months (SD = 31.6) in the SSNHL group and 39.3 months (SD = 31.0) in the control group. Age, sex, income, and region of residence were matched between the SSNHL and control groups (Table 1). The incidence of depression was higher in the SSNHL group (7.7% [332/4,328]) than in the control group (5.7% [993/17,312], P < 0.001). The CCI was different between the SSNHL and control I groups (P < 0.001).

SSNHL increased the HR of depression (adjusted HR = 1.29, 95% CI = 1.06–1.57; P = 0.010) (Table 2 and Fig. 1b). The SSNHL were also increased in depression with ≥ 4 times of treatments group compared to control II group (adjusted HR = 1.34, 95% CI = 1.08–1.68, P = 0.009, S1 Table).

In the subgroup analysis according to age and sex, middle-aged women showed high HRs for depression (adjusted HR = 1.46, 95% CI = 1.02–2.09, P = 0.042) (Table 4). The crude and adjusted HRs did not reach statistical significance in other age and sex subgroups.

**Table 4:** Subgroup analysis of crude and adjusted hazard ratios (95% confidence interval) of depression in sudden sensory neural hearing loss according to age and sex in study II.

Characteristics	HRs of depression			
	Crude†	P-value	Adjusted^†‡^	P-value
**Young men (20–39 years old, n = 2,315)**				
SSNHL	1.46 (0.52–4.04)	0.520	1.43 (0.51–4.06)	0.498
Control II	1.00		1.00	
**Young women (20–39 years old, n = 2,720)**				
SSNHL	1.45 (0.85–2.48)	0.176	1.35 (0.78–2.31)	0.284
Control II	1.00		1.00	
**Middle aged men (40–59 years old, n = 4,590)**				
SSNHL	1.45 (0.87–2.43)	0.152	1.27 (0.76–2.13)	0.362
Control II	1.00		1.00	
**Middle aged women (40–59 years old, n = 5,180)**				
SSNHL	1.55 (1.08–2.23)	0.017*	1.46 (1.02–2.09)	0.042*
Control II	1.00		1.00	
**Old men (60 + years old, n = 2,975)**				
SSNHL	1.51 (0.90–2.52)	0.119	1.32 (0.79–2.22)	0.295
Control II	1.00		1.00	
**Old women (60 + years old, n = 3,860)**				
SSNHL	1.23 (0.86–1.78)	0.263	1.21 (0.78–1.62)	0.544
Control II	1.00		1.00	

*Cox-proportional hazard regression model, Significance at P Co0.05.

*Cox-proportional hazard regression model, Significance at P Co0.05

^†^Stratified model for age, sex, income, and region of residence.

^‡^Adjusted model for Charlson Comorbidity Index.

## Discussion

Depression increased the risk of SSNHL (adjusted HR = 1.16, 95% CI = 1.02–1.31). On the other hand, SSNHL patients demonstrated an increased risk of depression (adjusted HR = 1.29, 95% CI = 1.06–1.57). In the subgroup analysis according to age and sex, these reciprocal associations between depression and SSNHL were consistent in the middle-aged women group. There was no study investigating the bidirectional association between depression and SSNHL in a single study, according to our knowledge. Moreover, few previous studies have explored the risk of SSNHL in depression according to age and sex.

In accordance with the present results, several researchers have demonstrated an association between depression and SSNHL^15,16^. However, most of the previous studies focused on the incidence of depressive symptoms or depression in SSNHL patients. The risk of depression was 2.17 times higher in SSNHL patients than in control participants in a retrospective cohort study ([95% CI] = 1.51–3.08, P < 0.001)^16^. Similarly, depressive symptoms were 2.04 times more likely to be present in SSNHL patients who have not yet recovered from SSNHL than in patients who recovered from SSNHL^17^. On the other hand, an elevated risk of SSNHL was reported in depressed patients. A prior study demonstrated an HR of 1.46 for SSNHL after adjustments for age, sex, income, region of residence, diabetes, hypertension, chronic kidney disease, and hyperlipidemia (95% CI = 1.10–1.93)^15^. The present study added to the previous findings by delineating the bidirectional association between SSNHL and depression. In addition, this study used multiple inclusion criteria of ICD-10 codes, pure tone audiometric examinations and treatment histories.

The impairment of auditory recognition function in depressed patients might contribute to the development of SSNHL. Decreased auditory processing could be linked to the onset of SSNHL in patients with depression. Auditory processing requires both peripheral auditory perception and central auditory processing. Thus, the impaired auditory processing and speech recognition might influence hearing function. For instance, impaired cognitive function was found to be related to hearing loss in previous studies^18,19^. Multiple cognitive functions, including executive function, verbal memory, and attention, are impaired in depressed patients^20^. Moreover, reduced activity, lack of drive and concern, and inability to plan could reduce the auditory input and processing of central auditory pathways^21^.

On the other hand, the decreased quality of life following SSNHL could increase the risk of depression. The stress from sudden hearing loss and accompanied symptoms of tinnitus or vertigo could impair the quality of life and influence the onset of depression. It was reported that annoying tinnitus and vertigo following SSNHL were the negative predictive factors for quality of life^22^. The impact of SSNHL on poor quality of life was highest in the aspect of mental functioning^23^. Another retrospective study demonstrated that the risk of depressive symptoms were higher in SSNHL patients who did not recover from SSNHL or complained of tinnitus^17^. In addition, un-recovered hearing loss resulted in the impairments in communication and social relationships, which was reported to be associated with cognitive impairment and depression^24^. In terms of neural function, hearing loss decreases the activities of the central auditory systems, which in turn increases the cognitive loads and disturb the emotional relativities^25^.

The undetected common pathologic causes, such as cardiovascular, endocrine, and autoimmune disorders, could mediate both depression and SSNHL. Moreover, the inflammatory cytokines C-reactive protein and interleukin-6 are elevated in depressed patients^26^. Increased peripheral inflammation could induce atherosclerosis and hamper the central nervous system, including the auditory nervous system. Diabetes could result in atherosclerotic changes and abnormal hormonal control. Hyperglycemia and high cortisol levels increase the risk of depression^27^. Indeed, the incidence of depression is 2–3 times higher in diabetic patients than in nondiabetic patients^28^. Moreover, the association between depression and diabetic control is bidirectional^27^. Depression increases the risk of hypoglycemia and hyperglycemia in diabetes patients and vice versa. Likewise, autoimmune disorders have been suggested to have a reciprocal relation with depression^29^. Because cardiovascular diseases, diabetes, and autoimmune disorders are also risk factors for SSNHL, the risk of SSNHL in depression could be attributed to these shared risk factors^30^.

Among the age and sex subgroups, the middle-aged female groups showed an increased risk of SSNHL among the depressed patients and increased risk of depression in SSNHL patients in this study. The high proportion of social activities and working positions in the middle-aged population compared to that in other age groups could increase the impact of depression on SSNHL in this age group. The sudden hearing loss and subsequent impaired communication might create more challenges and discomfort in the subjects with social activities and working positions. On the other hand, the low incidence of SSNHL in the young adult population could mitigate the association between depression and SSNHL in the present data. In the older population, causative factors other than depression, such as chronic diseases, could influence the development of SSNHL. A previous study reported that the association between depression and SSNHL was high in the younger age group (<60 years)^16^.

This study has several virtues in addition to the bidirectional analyses of the association between depression and SSNHL. The present study used a nationwide population cohort that was validated in a previous study^31^. All of Korea was included in the NHIS data, thus there were no missing participants. Control groups were randomly selected and matched for age, sex, income, and region of residence. Income and region of residence were matched and adjusted because socioeconomic status was one of the important determinants of medical care accessibility. Income was precisely investigated using the Korean NHIS data. In addition, possible confounders were comprehensively adjusted using CCI. For the accuracy of diagnoses, depression was evaluated ≥2 times by physicians, and SSNHL was classified based on the examination of pure tone audiometry and steroid treatment. However, the interpretation of the present results should involve the consideration of some limitations. Although numerous confounders were matched and adjusted in this study, there were still potential confounding factors, including stress factors, diet, physical activity, smoking and body mass index, that were not considered. The severity of depression and the treatment history of the depression patients could not be assessed using the NHIS data. To evaluate the association between depression and SSNHL, the depression patients with ≥4 times of treatment were analyzed for the reciprocal relation with SSNHL (S1 Table). The depression patients with ≥4 times of treatment also showed bidirectional association with SSNHL. Similarly, the severity and the prognosis of SSNHL were heterogeneous in the present study.

In conclusion, depression increased the risk of SSNHL in the adult. On the other hands, SSNHL increased the rate of depression in the adult population.

## Materials and Methods

### Study population and data collection

This study was approved by the ethics committee of Hallym University (2017-I102). The Institutional Review Board of Hallym University exempted written informed consent. All methods were performed in accordance with the guidelines and regulations of the ethics committee of Hallym University.

This national cohort study relied on data from the Korean Health Insurance Review and Assessment Service-National Sample Cohort (HIRA-NSC). The details of these data can be found in prior studies^32,33^.

### Participant selection

Out of 1,125,691 cases with 114,369,638 medical claim codes, we included participants who were diagnosed with depression. Depression was diagnosed based on the ICD-10 codes F31 (bipolar affective disorder) through F39 (unspecified mood disorder) between 2002 and 2013. Among them, the participants who visited clinics for depression ≥2 times (n = 68,019) following our previous studies^32,34^.

SSNHL was indicated by a diagnosis with the ICD-10 code H912. Among individuals with SSNHL, we selected participants who underwent audiometry exams (claim codes: E6931-E6937, F6341-F6348) and were prescribed steroids. Between 2002 and 2013, 5,244 of the SSNHL participants were included following our previous studies^35,36^.

#### Study I

**Figure 2:**
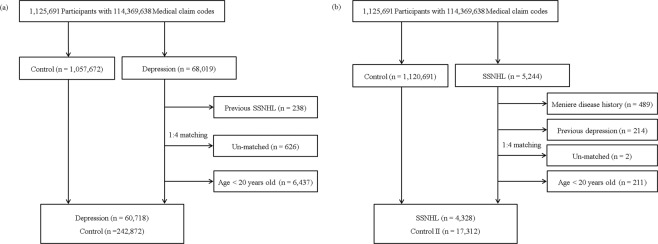
Schematic illustration of the participant selection process used in the present study. (**a**) Out of a total of 1,125,691 participants, 60,718 depressed participants were matched with 242,872 control participants according to age, group, sex, income group, and region of residence. (**a**) Out of a total of 1,125,691 participants, 4,328 SSNHL participants were matched with 17,312 control participants according to age, group, sex, income group, and region of residence.

In study I, the depressed patients and the control participants without history of depression were 1:4 matched 1:4. The control participants were extracted from the mother population (n = 1,057,672) and were matched for age, group, sex, income group, and region of residence as previously described^32,33^. The 238 participants who diagnosed SSNHL before the history of depression were excluded in the depression group. The 626 participants who had not enough matched control participants were excluded. The average period of follow-up was almost comparable between the depression group (71.9 months, standard deviation [SD] = 43.5) and the control group (73.2 months, SD = 43.3). The participants under 20 years of age were excluded because the prevalence of SSNHL in the young population was as low as 2.6% of whole age SSNHL patients, and the characteristics of SSNHL were different in these age groups (n = 6,437)^37^. Finally, the 60,718 depressed participants and 242,872 control participants were enrolled (Fig. 2). The occurrence of SSNHL in both depression and control I groups were analyzed. The SSNHL patients who had history of Meniere’s disease were excepted.

#### Study II

SSNHL patients and control II participants who were not diagnosed were 1:4 matched. Matching processes was similar with those of study I. The 489 participants with Meniere’s disease and 214 participants with history of depression were excluded. Two SSNHL patients were excluded due to the lack of control participants. The 211 participants with less than 20 years old were excluded. The average period of follow-up was almost comparable between SSNHL (55.8 months, SD = 38.4) and control groups (56.4 months, SD = 38.4). Lastly, the 4,328 SSNHL patients and 17,312 control II participants (Fig. 2b). The occurrence of depression in both SSNHL and control II participants were analyzed.

### Variables

Age, income, and region of residence were classified as previous studies^38^. The age groups were classified using 5-year intervals. The income groups were classified as 5 classes (class 1 [lowest income] − 5 [highest income]). The region of residence was divided into urban areas and rural areas.

The past medical history of each participant was evaluated using ICD-10 codes. The Charlson comorbidity index (CCI) was used as previous studies^39^.

### Statistical analyses

The demographic factors and other variables were compared between the depression and control groups using a chi-square test.

The hazard ratio (HR) of SSNHL for depression in study I, and the HR of depression for SSNHL in study II were analyzed using a stratified Cox proportional-hazards model. Crude and adjusted (CCI) models were calculated. The 95% confidence interval (CI) was evaluated. These analyses were stratified forage, sex, income, and region of residence. The Kaplan-Meier curve and Log rank test was conducted.

The participants were sub grouped according to age and sex (20–39 years of age, 40–59 years of age, 60 + years of age; men and women) because we thought that these associations may show different relations in the subgroups.

Two-tailed analyses were performed. The statistical significance was defined as P-values less than 0.05. The SPSS v. 21.0 (IBM, Armonk, NY, USA) and SAS version 9.4 (SAS Institute Inc. Cary, NC, USA) were used.

## Supplementary information

S1 Table.
