# Supplementary material for: Bidirectional relation between depression and sudden sensorineural hearing loss: Two longitudinal follow-up studies using a national sample cohort
Source: Sci Rep. 2020 Jan 30;10:1482. doi: 10.1038/s41598-020-58547-w (PMC6992784; doi:10.1038/s41598-020-58547-w)
Supplement: Supplementary file 1 — S1 Table. [file 41598_2020_58547_MOESM1_ESM.docx]

**Bidirectional relation between depression and sudden sensorineural hearing loss: Two longitudinal follow-up studies using a national sample cohort**

So Young Kim, MD^1^, Chanyang Min, PhD ^2,3^, Chang Ho Lee, MD,PhD ^1^, Bumjung Park, MD,PhD,^4^ Hyo Geun Choi,MD,PhD ^2,4*^

^1^Department of Otorhinolaryngology-Head & Neck Surgery, CHA Bundang Medical Center, CHA University, Seongnam, Korea

^2^Hallym Data Science Laboratory, Hallym University College of Medicine, Anyang, Korea

^3^Graduate School of Public Health, Seoul National University, Seoul, Korea

^4^Department of Otorhinolaryngology-Head & Neck Surgery, Hallym University College of Medicine, Anyang, Korea

**Running title:** Depression and Sudden sensory neural hearing loss

***Correspondence:** [pupen@naver.com](mailto:pupen@naver.com)

**Key words:** depression; hearing loss, sudden; mood disorder; bipolar disorder; risk factors; cohort studies

**S1 Table** Crude and adjusted hazard ratios (95% confidence interval) for SSNHL in depression treated ≥ 4 times (study I) and depression treated ≥ 4 times in SSNHL (study II)

| Characteristics | | | HRs | | | |
| --- | --- | --- | --- | --- | --- | --- |
|  | |  | Crude† | P-value | Adjusted†‡ | P-value |
| Study I | | | | | | |
|  | Depression | | 1.40 (1.23-1.60) | <0.001* | 1.19 (1.04-1.36) | 0.011* |
|  | Control | | 1.00 |  | 1.00 |  |
| Study II | | | | | | |
|  | SSNHL | | 1.48 (1.19-1.84) | 0.001* | 1.34 (1.08-1.68) | 0.009* |
|  | Control | | 1.00 |  | 1.00 |  |

* Cox-proportional hazard regression model, Significance at P < 0.05

† The model was stratified for age, sex, income, and region of residence.

‡ The model was adjusted for Charlson Comorbidity Index.
